# Dopamine-agonist responsive Parkinsonism in a patient with the SANDO syndrome caused by *POLG* mutation

**DOI:** 10.1186/1471-2350-14-105

**Published:** 2013-10-07

**Authors:** Monica Bandettini di Poggio, Claudia Nesti, Claudio Bruno, Maria Chiara Meschini, Angelo Schenone, Filippo M Santorelli

**Affiliations:** 1 Department of Neuroscience, Rehabilitation, Ophthalmology, Genetics, Maternal and Child Health, University of Genova and IRCSS Azienda Opedaliera Universitaria San Martino-IST, Largo Daneo 3-16132, Genova, Italy; 2 IRCCS Stella Maris Foundation, UOC Molecular Medicine, Neurodegenerative and Neuromuscular Diseases, Calambrone, Pisa, Italy; 3 Unit of Muscular and Neurodegenerative Disease, IRCCS G. Gaslini Institute, Genova, Italy

**Keywords:** POLG, Parkinsonism, Mitochondrial dysfunction, Ataxia, Progressive external ophthalmoparesis

## Abstract

**Background:**

Disorders of oxidative phosphorylation affects 1/5000 individuals and present heterogeneous involvement of tissues highly dependent upon ATP production.

**Case presentation:**

Here we present the case of a 48-year-old woman carrying a homozygous mutation (p.A899T) in mitochondrial polymerase gamma (*POLG*) and manifesting with a complex neurological phenotype including Dopamine-agonist responsive Parkinsonism.

**Conclusion:**

This case report is further evidence that mitochondrial dysfunction might play a role in Parkinson’s Disease pathogenesis and helps in identification of apparent mutation-specific clinical characteristics. Mutations in *POLG* should be looked for in cases of Parkinsonism, especially when multisystem neurological involvement is found.

## Background

The mitochondrial polymerase gamma (POLG) represents a major human disease gene and may account for up to 25% of all mitochondrial diseases, at least in UK and in Italy [1]. Among the possible clinical presentations, Alpers-Huttenlocher syndrome (AHS) in children and inherited progressive external ophthalmoparesis (PEO) in adults — either as the sole manifestation or in association with additional neurological features [2,3] — are the most common. Movement disorders are possible manifestations in AHS and have occasionally been described in adult PEO [4].

## Case presentation

Here we report on a patient carrying a homozygous mutation in *POLG* and manifesting with a complex neurological phenotype fitting the clinical diagnosis of SANDO (sensory ataxic neuropathy, dysarthria, and cophthalmoparesis) syndrome including Dopamine-agonist responsive Parkinsonism.

**Figure 1:**
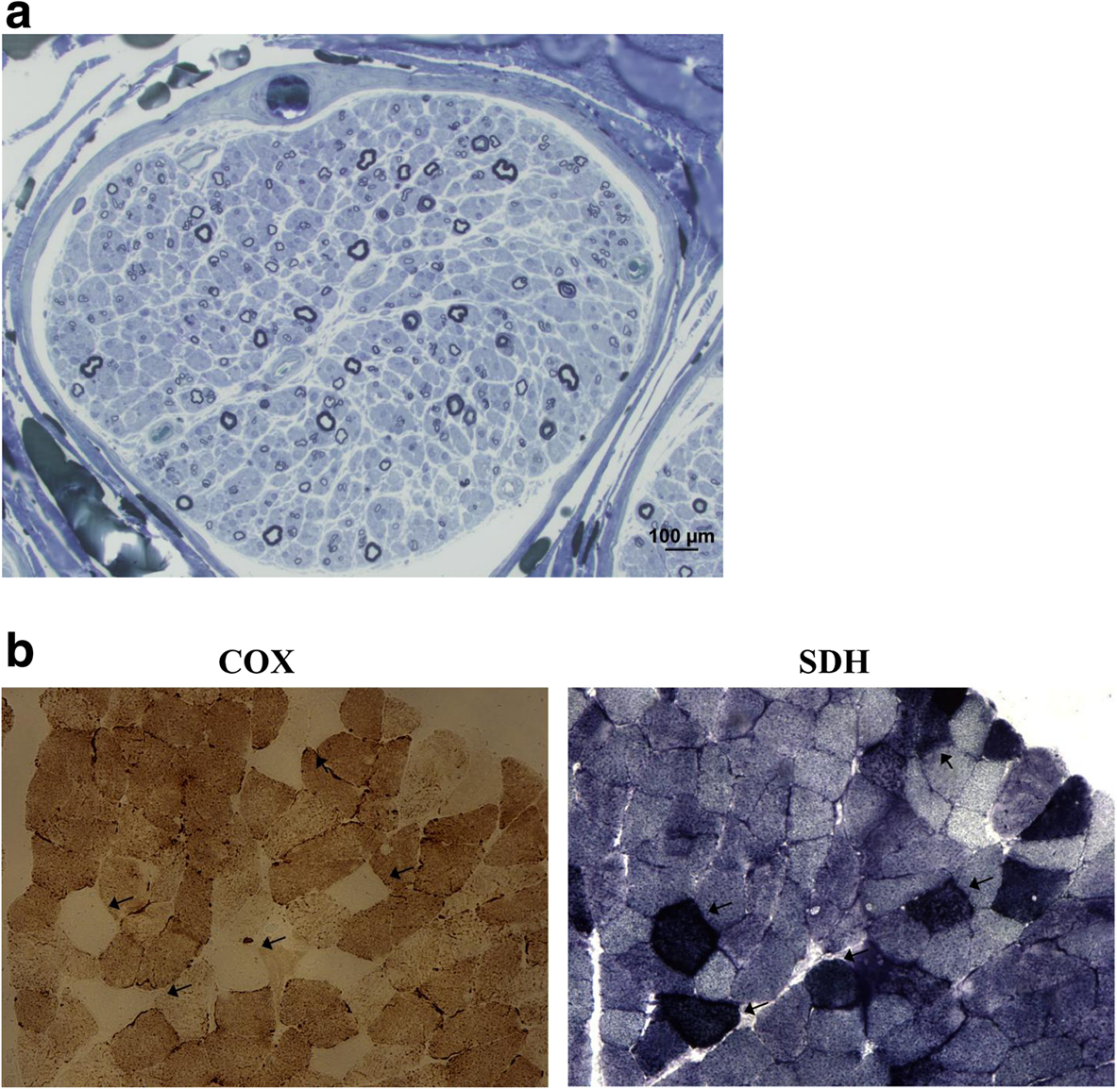
**Findings of nerve biopsy (semithin section in blue toluidine) and muscle sections stained for cytochrome c oxidase (COX) and succinate dehydrogenase (SDH) reactions. a**: Nerve biopsy shows loss of both small and large nerve fibers. **b**: Muscle biopsy shows several fibers with absence of COX activity (arrow) and marked mitochondrial proliferation, as shown by their strong SDH reaction.

**Figure 2:**
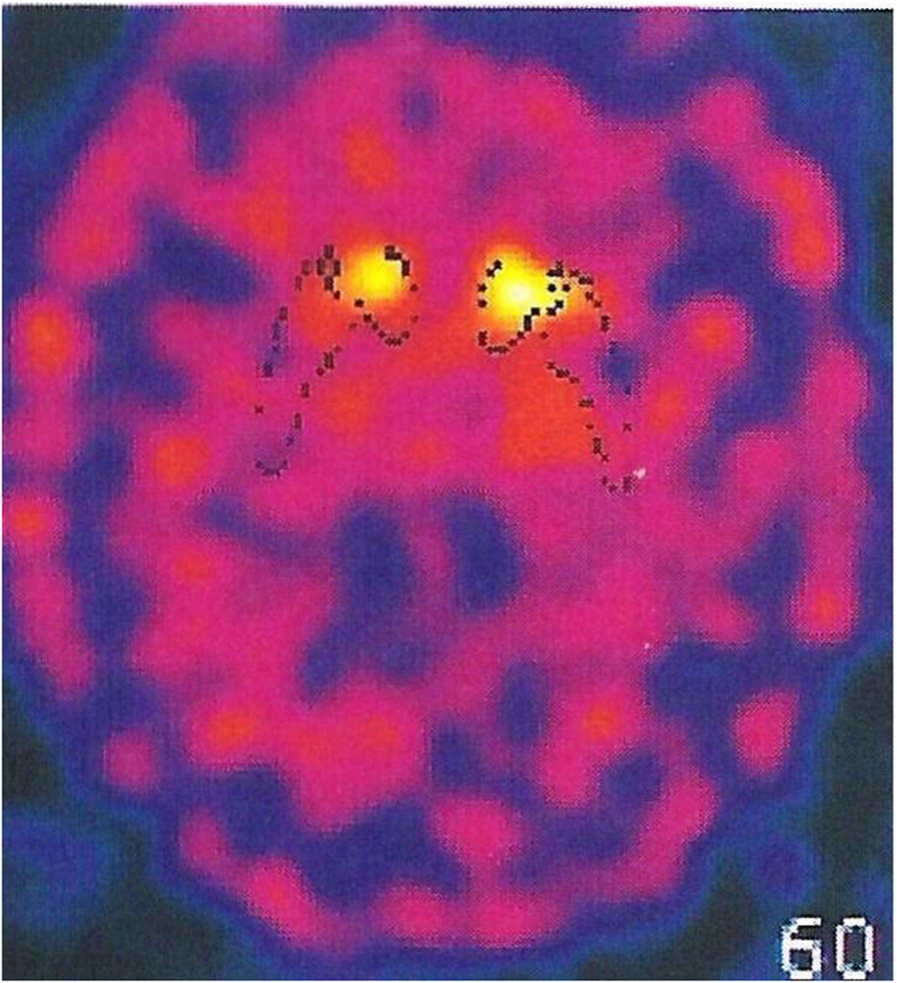
**I-FP-CIT SPECT imaging of the dopamine transporter.** The imaging of the dopamine transporter revealed reduced binding in both striata, more severe in the right putamen.

A 48-year-old Italian woman, born to non-consanguineous parents, and with a negative family history, was healthy until age 26 when she developed bilateral PEO and ptosis. At that age, electromyography showed myopathic features and a limb skeletal muscle biopsy was said to be compatible with mitochondrial myopathy, but whole mtDNA analysis was negative. At age 36, the proposita developed proximal and distal weakness in lower limbs, and sensory ataxia. A diagnosis of demyelinating sensory-motor neuropathy was considered on the basis of nerve conduction studies and sural nerve biopsy (Figure 1a). Anxiety-mood disorders became evident and treatment with SSRI was started (Fluoxetine 40 mg/day) with benefit. Histochemical and biochemical examination of a second muscle biopsy, using established methodologies for investigation of oxidative metabolism [5] showed “ragged blue”, cytochrome c oxidase negative muscle fibers (Figure 1b) and a partial biochemical reduction of activities of complex I and IV. At 47 years, the patient was referred to our attention because of onset of resting and attitudinal hand tremor and worsening in gait and posture. Neurological examination showed PEO, bilateral ptosis, signs of sensorimotor neuropathy with ataxic gait and positive Romberg sign, head and limbs tremor plus rigidity. Unified Parkinson’s Disease Rating Scale-UPDRS motor score was 39. Cognition was mildly affected upon MMSE examination, and anxiety and obsessive disorder were evident. Creatine kinase levels were 217 U/L (normal < 168) and myoglobin was 360 U/L (normal <75). A brain MRI showed mild cortical atrophy. I-FP-CIT SPECT imaging of the dopamine transporter revealed reduced binding in both striata (Figure 2). Dopamine agonist treatment (Pramipexole R.P. 0.52 mg/day) was started with improvement in tremor and ambulation (UPDRS III = 29). After a 10-month follow up, the patient remains on treatment with Pramipexole R.P. (1.05 mg/day) and Duloxetine (60 mg/day) with a stable neurological condition.

The 82-year-old mother and two of the four living sisters, aged 57 and 45 years, had a normal neurological examination. The father of the proposita had died at the age of 72 because of myocardial infarction but he was referred to be free of neurological complaints.

**Figure 3:**
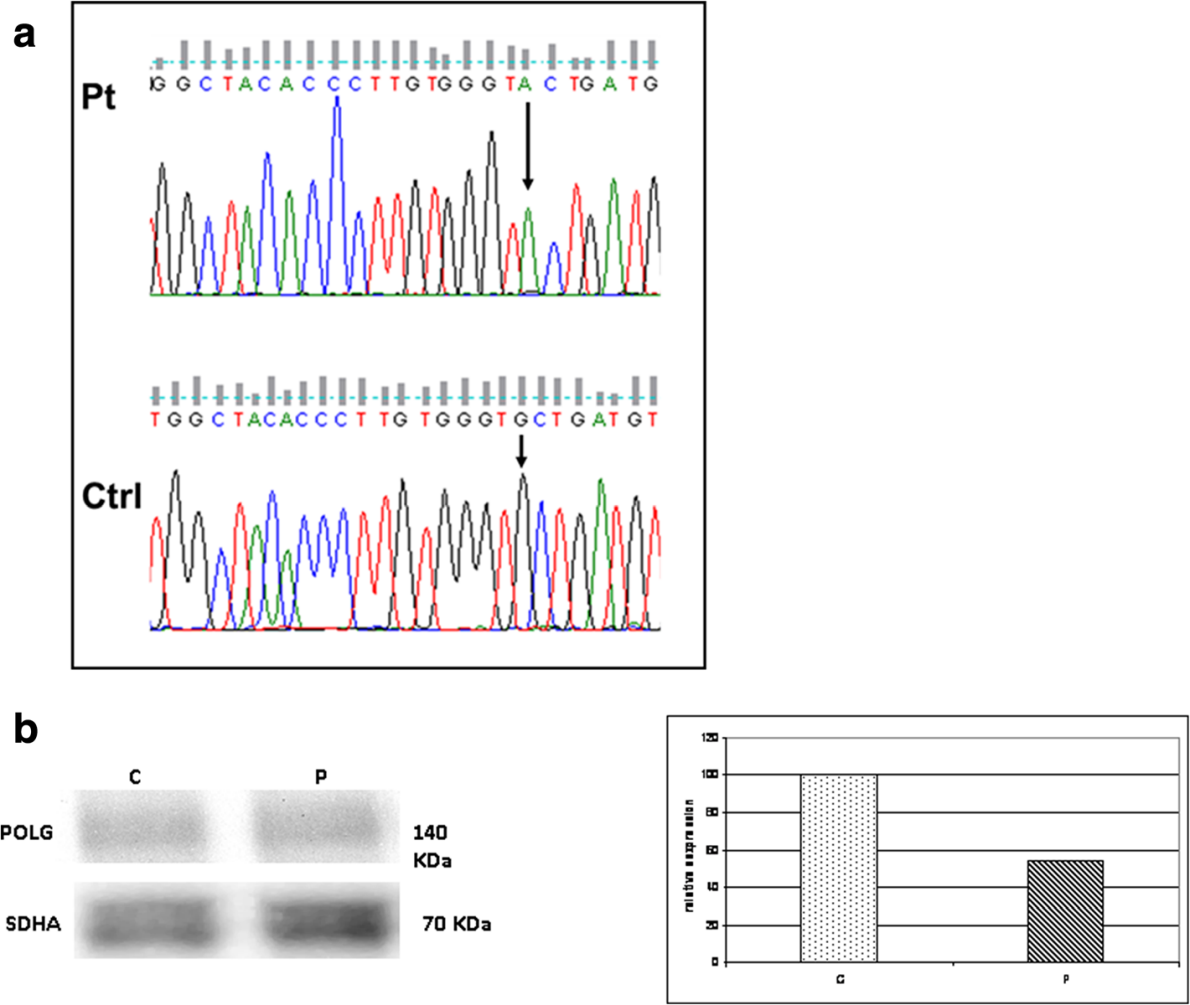
**Electropherogram of *POLG (a) *and Western blot and densitometric analysis of POLG protein (b) (1:100, ACRIS antibodies, Germany) in the patient and a control. a**: The electropherogram with the region of the exon 17 of *POLG* flanking the homozygous mutation (c.2665G>A) identified in the patient. The electropherogram of a control is also shown. Arrow indicates the mutation. **b**: About 50 μg of skeletal muscle homogenate were loaded in each lane. POLG content was normalized using a monoclonal SDHA antibody (1:500, Mitosciences, USA). Levels of expression in the patient appear reduced (about 45% of control sample). A representative experiment is shown.

Having obtained written informed consent, genomic DNA was extracted from peripheral blood of the patient and her living relatives using the MagNa Pure System (Roche) and the whole coding region and flanking intron-exon boundaries of *POLG* were directly sequenced using the BigDye 3.1 chemistry (Applied Biosystems, Foster City, CA). In the proposita we identified a homozygous c.2665G > A/p.A889T change (Figure 3a). The mutation was heterozygous in her mother and two sisters and led to a reduction of the protein in skeletal muscle homogenate (Figure 3b). No mutations in other genes associated with multiple mtDNA deletions were detected [6]. There was no mtDNA depletion in muscle.

## Conclusion

The present report offers two main considerations. The patient we described combines the clinical features of SANDO syndrome complicated with late-onset Parkinsonism and mood disorder. In previous years, co-existence of Parkinsonism and *POLG* mutations has been described, suggesting that mitochondrial dysfunction might play a role in the pathogenesis of PD [7]. Our case report is further evidence that abnormal oxidative metabolism and loss of mtDNA integrity might be implicated in similar conditions. Whilst is evident that *POLG* does not represent a frequent etiology in PD-like syndromes, it seems not too speculative to hypothesize that alterations in mtDNA fidelity and subsequent impaired protein synthesis likely compromise mitochondrial bioenergetics, dynamics, transport, or their combination, in dopaminergic neurons [8]. Similar to other cases of “mitochondrial parkinsonisms”, our patient had benefit with antiparkinson drugs, underling the importance of a correct diagnosis [4,9-12].

A further consideration emerges from the evidence of an additional association between the *POLG* p.A899T variant and SANDO syndrome. The p.A899T has initially been described in compound heterozygosity [13,14] and frequently associated with the clinical triad of sensory ataxic, ptosis, and PEO together with a mood disorder and a movement disorder such as Dopamine-agonist responsive parkinsonism. In the face of an ever increasing, pleiomorphic array of features associated with mutations in *POLG*[15], identification of apparent mutation-specific clinical characteristics might facilitate molecular confirmation in complex patients, prevent possible co-morbidities, and permit to adopt effective symptomatic therapies.

## Abbreviations

POLG: Mitochondrial polymerase gamma; AHS: Alpers-Huttenlocher syndrome; PEO: Progressive external ophthalmoparesis; SANDO: Sensory ataxic neuropathy, dysarthria, and ophthalmoparesis; mtDNA: mitochondrial DNA; MMSE: Mini Mental State Examination; UPDRS: Unified Parkinson’s Disease Rating Scale.

## Competing interests

All authors declare that they have no competing interests.

## Authors’ contributions

MBDP has made substantial contributions to conception, design and coordination of the study, has been involved in drafting the manuscript and has given final approval of the version to be published. CN has been involved in drafting the manuscript, carried out the molecular genetic studies and has given final approval of the version to be published. MCM has been involved in drafting the manuscript, carried out the molecular genetic studies and has given final approval of the version to be published. CB carried out the examination of muscle biopsy and has made substantial contributions to acquisition of data and has given final approval of the version to be published. AS has been involved in drafting the manuscript and revising it critically for important intellectual content and has given final approval of the version to be published. FMS has made substantial contributions to conception, design and coordination of the study, has been involved in drafting the manuscript and has given final approval of the version to be published.

## Pre-publication history

The pre-publication history for this paper can be accessed here:

http://www.biomedcentral.com/1471-2350/14/105/prepub
